# Folic acid grafted mixed polymeric micelles as a targeted delivery strategy for tamoxifen citrate in treatment of breast cancer

**DOI:** 10.1007/s13346-023-01443-3

**Published:** 2023-10-31

**Authors:** Mohamed Nasr, Fahima Hashem, Mohammed Teiama, Norhan Tantawy, Raghda Abdelmoniem

**Affiliations:** 1 Department of Pharmaceutics and Industrial Pharmacy, Faculty of Pharmacy, Helwan University, Cairo, 11790 Egypt; 2 Department of Pharmaceutics, Faculty of Pharmacy, Delta University for Science and Technology, Gamasa, 11152 Egypt; 3 Department of Pharmaceutics and Industrial Pharmacy, Faculty of Pharmacy, Galala University, Attaka, 43713 Suez Egypt

**Keywords:** Tamoxifen citrate, Folic acid, Pluronic copolymers, Mixed polymeric micelles, Antitumor activity, Targeting breast cancer

## Abstract

The objective of this study was to develop folic acid (FA) grafted mixed polymeric micelles loaded with Tamoxifen citrate (TMXC) to enhance its antitumor activity in breast tissues. The conjugated folic acid Pluronic 123 (FA-P123) was prepared using carbonyl diimidazole cross-linker chemistry and confirmed using FTIR and ^1^HNMR. TMXC-loaded P123/P84 (unconjugated) and TMXC-loaded FA-P123/P84 (conjugated) micelles were examined for encapsulation efficiency, particle size, surface charge, in vitro drug release, cytotoxic effect, and cellular uptake by a breast cancer cell line. The conjugated TMXC-loaded micelle exhibited a nanoparticle size of 35.01 ± 1.20 nm, a surface charge of—20.50 ± 0.95 mV, entrapped 87.83 ± 5.10% and released 67.58 ± 2.47% of TMXC after 36 h. The conjugated micelles exhibited a significantly higher cellular uptake of TMXC by the MCF-7 cell line and improved in vitro cytotoxicity by 2.48 folds compared to the TMXC-loaded unconjugated micelles. The results of in vivo studies indicated that TMXC-loaded FA-P123/P84 has a potential antitumor activity, as revealed by a significant reduction of tumor volume in tumor-bearing mice compared to TMXC-loaded unconjugated micelles. In conclusion, the obtained results suggested that conjugated FA-P123/P84 micelles could be an encouraging carrier for the treatment of breast cancer with TMXC.

**Graphical abstract:**

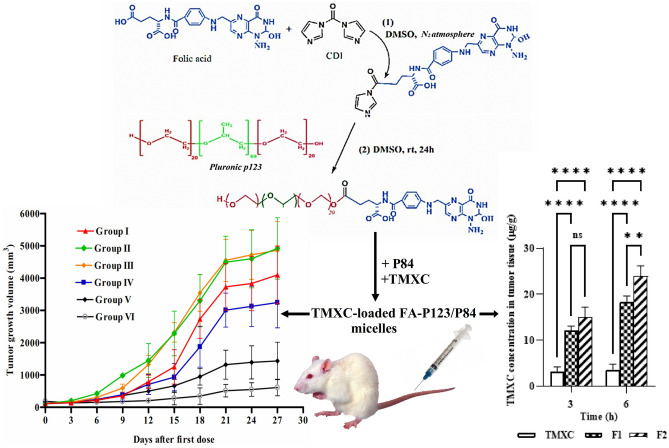

## Introduction

Breast cancer represents the most common type of malignancy among females, accounting for one-third of all types of malignancies as well as showing a remarkable annual expansion with greater related death cases than the other types of tumors in women [1]. Although it seriously threatens the woman’s life, early screening and diagnosis in the primitive stages might facilitate the treatment story [2]. Until now, Tamoxifen, Paclitaxel, Methotrexate, Adriamycin, and Docetaxel have been approved as chemotherapeutic agents for breast cancer treatment. Nevertheless, most of these chemotherapeutics confront a high level of resistance for their efficacy with remarkable side effects including their poor solubility in the body and the absence of precise targeting ability [3]. Furthermore, the failure of drug action and tumor deterioration resulted from multidrug resistance (MDR) owing to prolonged treatment [1]. Therefore, the fabrication of novel delivery systems that maximize drug loading, increase drug solubility, enhance permeability and retention effect (EPR), and achieve target effects with optimum stability is necessary to overcome drug drawbacks at other sites [4].

Among the most popular chemotherapeutic drugs, Tamoxifen citrate (TMXC) was utilized for all stages of breast cancer. It acts as an antagonist that prevents activation of the receptor in the tissue by endogenous estrogens. However, numerous side effects comprise extreme drug resistance, low therapeutic index, poor solubility, and other body disorders; hot flashes, vaginal bleeding, insomnia, vaginal pruritus, fluid retention, and tumor inflammation [5]. Therefore, utilizing a novel nano platform employing biocompatible polymers like polymeric micelles (PMs) as a promising carrier was the better choice. PMs have raised distinct interest as nano-sized carriers due to their exceptionally advanced properties; high loading capacity with good solubilization, extended drug release, and active targeting drug delivery via suitable ligands with abundant stability [6]. Moreover, they can improve drugs’ bioavailability and their desired biopharmaceutical and pharmacokinetic features in the treatment of tumors [7]. Additionally, their labeling allows drugs to accumulate in tumor tissue, elevating cellular uptake via enhanced retention and permeation mechanisms through the leaked blood vessels in compromised tissues [8].

Pluronic (poloxamer) copolymers are most used owing to their unique characteristics, increasing drug solubility, and stability with lacking immunogenicity, antigenicity as well as toxicity. Moreover, they demonstrated to increase the therapeutic effect of drugs as they can inverse the multi-drug resistance (MDR) effect in cancer therapy by inhibiting drug efflux transporters on the surface of cancer cells. However, single-surface surfactant pluronic micelles displayed some drawbacks such as low cargo loading, limited stability, and large particle magnitude. Therefore, in our previous study [9], a mixture of Pluronic polymers (P123 and P84) was fabricated as a micellar system at low concentrations (90:10 w/w) which encapsulated a large amount of TMXC with a narrow size and ideal kinetic stability. Nevertheless, Pluronic micelles as a promising potential carrier for chemotherapeutic agents, lack targeting action to breast cancer receptors and subsequently their selectivity. Hence to improve the micelles prepared before, in this study we stated a novel polymer-conjugate in mixed polymeric micelles fabrication.

Folic acid (FA) was elected as a desirable choice for targeting action owing to its receptors which presented in the great majority of breast cancer cells while being little distributed in normal healthy tissue [10]. FA is a non-immunogenic, naturally occurring vitamin B with a small molecular weight that is converted inside the body into folate which exhibits a great tendency for the receptor [11]. Owing to its numerous investigations in PMs Conjugation in targeting anti-tumor drugs as well as genes, FA covalently conjugated to the block copolymer P123 in our study was selectively taken up into cells overexpressing FA receptors via receptor-mediated endocytosis, attain promise active targeting. [12, 13].

Bearing in mind these characteristics, our work was carried out to explore the efficacy of conjugated polymeric micelles (FA-P123/P84) as a carrier for the systemic transfer of TMXC cargo. The conjugated micelles were investigated for their encapsulation efficiency (EE), particle size (PS), surface charge (ZP), in vitro drug release, and cellular uptake to examine the success of system fabrication as well as targeting efficacy. Furthermore, MCF-7 cell line and tumor-bearing mice were utilized for the evaluation of conjugated micelles cytotoxicity and in vivo antitumor efficacy.

## Materials and methods

### Materials

Tamoxifen citrate was generously gifted by Medical Union Pharmaceuticals (98.9% purity, Abu Sultan, Ismailia, Egypt). Pluronic P84^®^, M.wt. 4200 g/mol (P84) and Pluronic P123^®^, M. wt. 5700 g/mol (P123) were kindly gifted from Badische Anilin und Sodafabrik BASF SE (Ludwigshafen, Germany) without additional purification. Folic acid (M.wt. 441.4 g/mol), 1,1 carbonyl diimidazole, M.wt. 162.15 g/mol (CDI), acetonitrile (HPLC grade), methanol, and anhydrous dimethyl sulfoxide (DMSO) (HPLC grade) were bought from Sigma-Aldrich, Germany. RPMI 1640 cell line growth media was supplied by Lonza Bioscience (Biological Products Company), Morristown, NJ 07960, USA). The MCF-7 cell line was supplied by The National Cancer Institute (NCI), Egypt. The remaining chemicals of high analytical grade were purchased from El-Nasr Pharmaceutical Chemicals Co (Cairo, Egypt).

### Animals

About 72 healthy adult female Swiss Albino mice were provided by the National Cancer Research Center (NCRC, Cairo, Egypt) with average weight (20–25 g). Animal handling before and during the experimental work was carried out according to Animal Research Ethical Committee approval no 17A2022 (Animal Experiment of Pharmaceutical Science Committee, Faculty of Pharmacy, Helwan University, Cairo, Egypt).

### Fabrication of folate conjugated Pluronic 123 (FA-P123)

Folate conjugated P123 complex was developed using CDI as a cross-linker based on the method progressed by [14] with some modifications. Concisely, to activate FA carboxylic moieties; CDI and FA at a ratio of 1:1 w/w were dissolved in 6 mL anhydrous DMSO with continuous stirring overnight in the dark under a nitrogen atmosphere. After FA activation, P123 (0.1 mmol) was added and allowed to interact for 24 h at room temperature under dark conditions. By the end of the experiment, the yield was immersed in DMSO for 2 days using a dialysis bag (Spectra Millipore MWCO 3500), followed by further 2 days against deionized water [15]. The obtained FA-P123 conjugate was then lyophilized and kept at -20 °C. Proton NMR (^1^HNMR) spectra and Fourier transform infrared spectroscopy (FTIR) of pure P123, FA, and FA-P123 conjugate were analyzed to confirm the conjugation of FA onto P123.

### Characterization of prepared conjugation (FA-P123)

#### FTIR spectra

To inspect the formation of the FA-P123 conjugate, FTIR spectra for P123, pure FA, and FA-P123 were obtained using a spectrophotometer (JASCO FTIR- 8400, Japan). Each sample was mixed well individually with potassium bromide (KBr) obtaining translucent discs for scanning. The materials were screened in the range of 4000–400 cm^−1^ to attain respective spectra [16].

#### ^1^HNMR spectra

^1^HNMR spectra of FA, P123, and FA-P123 conjugate were attained by NMR spectrophotometer (Bruker 400, Switzerland) to confirm FA-P123 conjugate synthesis. DMSO was used as a solvent for FA, while deuterated water (D_2_O) was the suitable solvent for P123 and FA-P123 to reveal their chemical structures. Each sample solution was transferred to NMR tubes and shifted chemical peaks were detected as parts per million (ppm) [11].

#### CMC measurement

The CMC values of the P123-P84 and FA-P123/P84 mixed polymeric micelles were examined in deionized water as previously described by [9]. Briefly, the standard KI/I_2_ solution was prepared by dissolving iodine and potassium iodide in 100 ml of deionized water at a ratio of 1:2 w/w, respectively. The CMC was determined using different concentrations ranging from 0.00001–0.1% for polymer solutions linked with 25 µL of standard solution. The prepared mixtures were incubated in dark conditions at ambient room temperature for 12 h, then, the absorbance of all samples was screened at 366 nm. Experimental work was carried out in triplicate and the mean absorbance was drawn versus the polymer concentration logarithmic values. The absorbance increase against the increase of the polymer concentration was followed to detect the CMC at the point of sharp change in absorbance.

## Preparation of TMXC-loaded mixed polymeric micelles

TMXC-loaded P123/P84 (F1) and TMXC-loaded FA-P123/P84 (F2) were obtained by the thin film hydration method as previously reported by [17] with some modifications. Concisely, a mixture of TMXC and P123/P84 or TMXC and FA-P123/P84 in a weight ratio of TMXC to the mixed Pluronic polymers of 1 to 50 was dissolved in 10 ml methanol. The ratio of P123 to P84 in F1 or FA-P123 to P84 in F2 was 9:1 w/w in both formulations. The mixture was then evaporated under vacuum by a Rota evaporator (IKA, HBIO basic, Model RV10B S99, Deutschland, Germany) at 200 rpm and 50 °C until the thin film was obtained. The flask was kept overnight at room temperature to confirm solvent evaporation, subsequently, the film was hydrated for 30 min with 10 ml of deionized water at 50 °C. Finally, the obtained dispersion was filtered through a 0.22 µm cellulose acetate membrane and was lyophilized in the presence of 5% mannitol as a cryoprotectant. The free polymeric dispersions (P123/P84 and FA-P123/P84) were prepared by the same procedure without the addition of TMXC.

## Characterization of TMXC-loaded mixed polymeric micelles

### Encapsulation efficiency (EE) %

The amount of free un-entrapped TMXC was estimated after dialysis by UV–VIS spectrophotometer at 277 nm [9]. The obtained micellar solution was diluted with anhydrous methanol to a certain concentration before UV screening. The loaded (DL%) and entrapped (EE%) drug were calculated by the following equations.

$$\begin{aligned}\% \mathrm{\;DL}=&\,\mathrm{(Weight\;of\;drug\;in\;micelles}\,\\&/\,\mathrm{Total\;weight\;of\;copolymer\;and\;drug\;in\;micelles)}\times100\end{aligned}$$

$$\begin{aligned}\%\;\mathrm{EE}=&\,\mathrm{(Amount\;of\;drug\;present}\\&/\,\mathrm{Total\;amount\;of\;added\;drug)}\times100\end{aligned}$$

### Particle size, PDI, and surface charge

The average particle size, surface charge, and polydispersity of TMXC-loaded mixed polymers were scrutinized using a Malvern Zetasizer (Malvern Instruments/Worcestershire, UK) based on the dynamic light scattering principle. Experimental work was performed in triplicates after dilution with deionized water at an angle of 90**°** at 25 ± 0.5 ºC.

### In vitro drug release

The drug release from the formulae F1 and F2 was investigated in phosphate buffer (pH = 7.4) including 0.1% (w/v) of Tween 80 using a dialysis technique [18]. A volume of micellar dispersion equivalent to 3 mg TMXC was poured into a pre-soaked dialysis bag (MWCO: 12–14 kDa, Livingstone, NSW, Australia) and immersed into a 40 ml release medium with a stirring rate of 100 rpm at 37 °C ± 0.5 °C. At scheduled time intervals, 1 ml aliquot of the release media was withdrawn and replaced immediately with a fresh release medium to keep the sink conditions [19]. The cumulative percentage of the released drug was quantified spectrophotometrically at λ max 279.50 nm. The obtained results were compared with pure TMXC release performed in the same conditions. To estimate the release kinetics, the release data were checked with different kinetics models representing (zero-order, first-order, Higuchi diffusion equations, and Korsmeyer–Peppas models). Correlation coefficients (R^2^) values were analyzed for the highest regression value and compared for election the most appropriate kinetics model that best fits the data [20, 21].

### Effect of storage

The effect of storage on TMXC-loaded FA-P123/P84 (F2) was conducted by storage in a desiccator for 3 and 6 months at 25 °C. During this period, particle size, PDI, zeta potential, and EE% were inspected. Student’s t-test was applied to detect any statistical significance at *P* < 0.05 using SPSSVR software version 22.0 (SPSS Inc., Chicago, IL).

### In vitro cytotoxicity and cellular uptake studies

#### In vitro cytotoxicity

Cell culture was carried out in aseptic conditions. The breast cancer cell line (MCF-7) was incubated at 95% humidity, 5% CO_2_, and 37 °C temperature. It was cultivated in T-75 tissue culture (RPMI 1640 media) containing 1% antibiotics (Streptomycin, Amphotericin, Penicillin) and 10% fetal bovine serum. Cells were sub-cultured once they had reached 70–80% confluence using fresh media, whereas, the cells were watched for the appropriate stage of exponential growth (sub-confluence) [22].

The cytotoxicity of F1 and F2 on human breast cancer cells (MCF7) was conducted using MTT assay for 24 h [23]. Briefly, cells were seeded in a 96-well plate with a cell density of 10,000 cells /well in culture flasks using RPMI 1640 supported by 10% FBS, and 100 μL of penicillin G, streptomycin antibiotic mixture at 37 °C. The cells were treated with different concentrations series that were ranging from 1 to 20 μg/ml and incubated for 72 h. After an incubation period, the media was replaced with 120 μL of MTT solution with a concentration of 5 mg/mL for a further 4 h at 37 °C. Finally, the media was withdrawn and replaced with 200 μL of DMSO to dissolve formazan crystals [24, 25]. All plates were scanned with a plate reader (ChroMate-4300, FL, USA) at 570 nm. At least 3 replicates were carried out for each testing condition. The cell death and viability percentage in all plates were estimated with the equation:

$$\begin{aligned}\%\;\mathrm{Cell\;viability}=&\mathrm{\;(Absorbance\;of\;treated\;cells}\\&\mathrm{/\;Absorbance\;of\;treated\;cells)}\times100\end{aligned}$$

#### Cellular uptake study

MCF-7 cancer cells were seeded at a cell density of 5 × 10^4^ cells/well in 96-well plates, in culture medium (200 μl of RPMI 1640) and incubated for attachment overnight. Cells were then treated with free TMXC, F1, and F2 containing an equivalent amount of drug (5 μg/ml) and incubated at 37 °C under 5% CO_2_. The plates were washed with saline (PBS, pH 7.4) three times after removing growth media of 3 and 6 h incubation [26]. The collected materials and washing saline were inspected for TMXC concentration using LC/MS/MS (Agilent Technologies, Inc., Santa Clara, CA). LC–MS assay and a calibration curve of TMXC were carried out previously in our investigations [9]. Negative controls with untreated cells of the same density were employed. To explore the cellular uptake, the decrease in the external concentration of non-taken TMXC / TMXC micelle was calculated using the following equation.

$$\% \; \text{Cumulative intracellular TMXC} = [(\text{C}_{0}-\text{C}_\text{exa t}) / \text{C}_{0}]\times 100$$

where C_**0**_ is the initial concentration of TMXC added and C_**exa** t_ is the extracellular un-taken concentration of TMXC at time = t.

### In vivo antitumor effect on animal model

The experimental animal protocol was authorized by the committee of animal experimentation ethics of the Faculty of Pharmacy, Helwan University (Approval no: 17A2022). All mice (72 healthy adult female Swiss Albino) were housed in Makrolon IV polycarbonate cages and given free access to water and diet pellet food during the experiment under 55% humidity and 22 °C temperature. Ehrlich carcinoma cell line was injected subcutaneously into the thigh of each mouse using a 23-G needle to induce tumors [27]. After 15 days, intense solid tumors with an average mass volume of more than 100 mm^3^ were observed. The tumor-bearing mice were randomly allocated into six groups (12 mice each). The first group received an isotonic saline as untreated control. Free P123/P84 and Free FA-P123/P84 micelles were used for the 2^nd^ and 3^rd^ groups, respectively. While the remaining groups; IV, V, and VI received TMXC, F1, and F2 dispersions, respectively. A volume of 250 µL of each treatment was injected through the tail vein. For drug-treated groups, the concentration of TMXC was 1 mg/ml, and the dose was maintained to be 10 mg/kg of body weight. Four consecutive doses were administered each week for all groups. The weight of mice and volume of tumor were recorded twice per week.

#### Evaluation of tumor volume (V) and % tumor growth inhibition (% TGI)

Tumor volumes were estimated by measuring two different perpendicular diameters of the tumor mass using a digital caliper. The small diameter was termed as width with the symbol (a) and the larger diameter was termed as length with the symbol (b). Both diameters were observed and recorded for mass tumors to calculate the development in tumor volume and calculate the inhibition of tumor growth as a response for treatment. Tumor volume was calculated as follows [28]:

$$\text{Volume = }\text{(a}^{2}\times\text{b)/ 2}$$

Also, % tumor growth inhibition (% TGI) is calculated as follows.

$$\%\;\text{TGI} = 100 - (\text{T/C}\times 100)$$

where T is the mean relative tumor volume (RTV) of the treated group and C is the RTV of the control group. RTV of any group is calculated by the following equation [29]:

$$\text{RTV = Vx/Vi}$$

where Vx is the tumor volume at the end of the experiment and Vi is the tumor volume at the start day of treatment.

#### TMXC biodistribution in tumor tissues

At the end of the study, the mice were sacrificed; tumor samples were collected for TMXC quantification. One gram of tumor was weighed and rinsed instantaneously with phosphate buffer saline followed by homogenization (tissue-homogenizer, Wilmington, USA) at 4000 rpm for 3 min using 5 ml of 50 mM Tris–HCl buffer. After removing TMXC from the tumor homogenate employing acetonitrile, samples were vortexed for 30 s [30]. Finally, samples were centrifuged for any aggregated proteins for 10 min at 4000 rpm and the obtained supernatant was further filtered through a micro-syringe filter of pore size 0.22 mm. The drug level in supernatant was estimated using an HPLC–MS analyzer (Thermo Scientific Accela, USA) as previously described in our published work [9].

### Histopathological assay

To evaluate the histopathological changes in tumor tissues after TMXC treatments, tumor samples were collected from groups I, IV, V, and VI and stored in 10% formalin phosphate buffer saline for one day. H&E staining essay was achieved by dehydrating the tissues with serial dilution of ethanol and xylene, then embedding for fixation in paraffin wax at 56 °C for another 24 h. Paraffin sections. (4-μm thick) were gathered on glass slides, dewaxed and stained with H&E stain, and examined by light electric microscope (Olympus, CX41, Japan) [31].

## Statistical analysis

Data analysis was carried out using GraphPad in the Stat program (version 3). Results were calculated and represented as a mean value ± standard deviation. One-way (ANOVA) test paired, and un-paired student t-test were used for the estimation of statistically significant difference at *P* < 0.05 as a minimal level of significance.

## Results and discussion

### Synthesis of FA-P123 conjugate

**Fig. 1:**
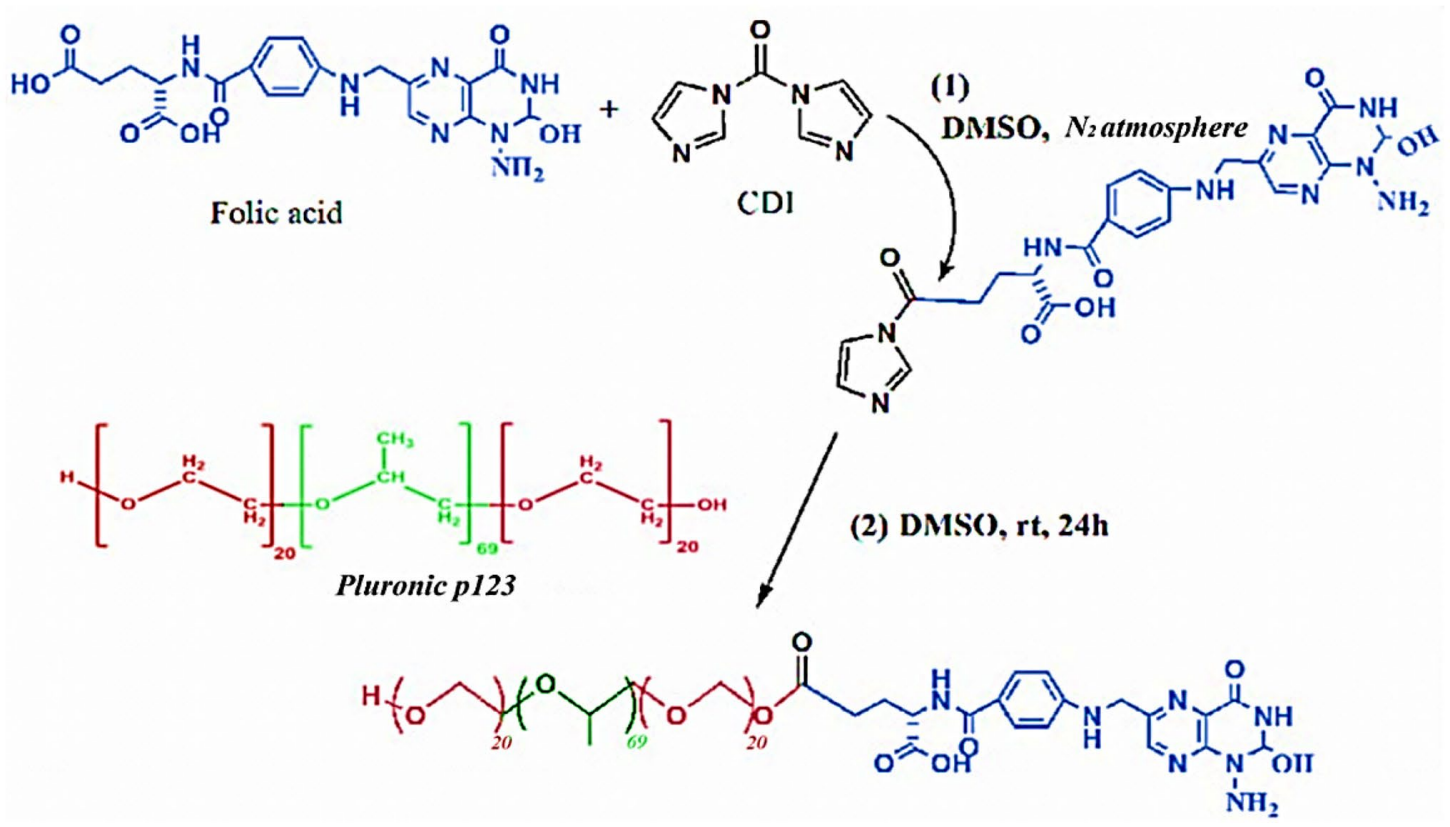
Synthetic route of the FA-P123 conjugate

In this study, the FA-P123 conjugate was synthesized successfully according to the synthetic route that is illustrated in Fig. 1. Folic acid was conjugated to Pluronic P123 in accordance with the results of the prior study [14]. Primarily, one of the carboxylic groups of folic acid was activated by reacting with one of the carbonyl di-imidazone group of CDI, as revealed in the first reaction. This step was followed by the interaction of the hydroxyl group of P123 with the remaining carbonyl di-imidazone group of CDI, as revealed in the second step. This reaction was achieved with a molar ratio of 1:1.1:5 between folic acid, CDI, and P123 which assures that at least one of the hydroxyl groups of P123 was conjugated to the folic acid.

### Characterization of folate conjugated Pluronic (FA-P123)

#### FTIR spectra

**Fig. 2:**
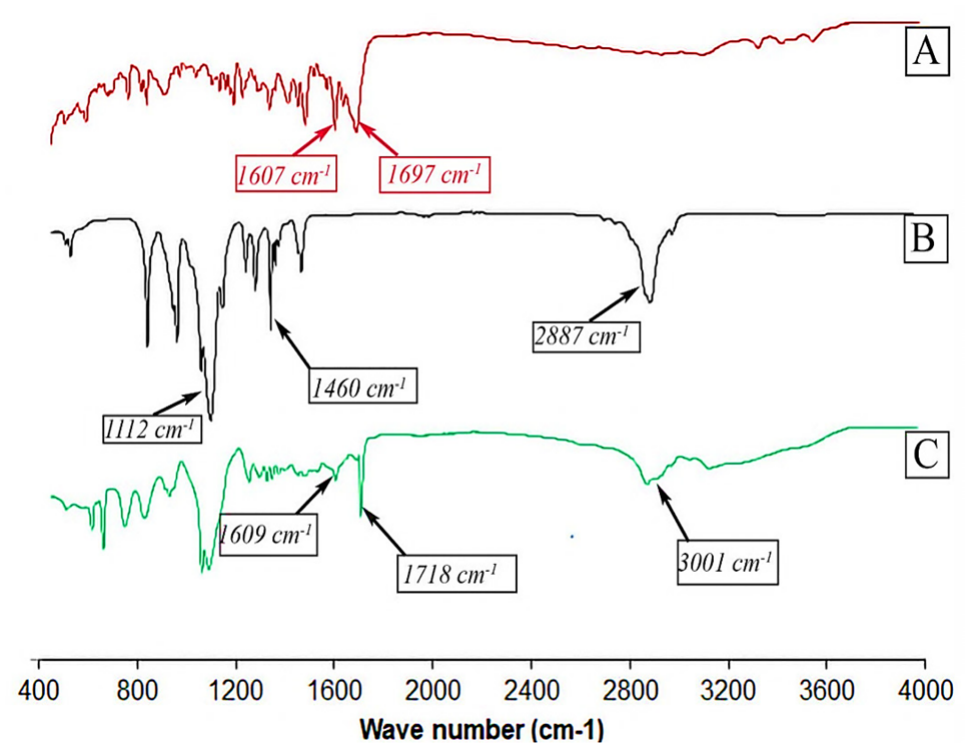
FTIR spectra of FA (**A**), P123 (**B**), and FA-P123 (**C**)

To characterize FA-P123 and explore intermolecular interaction in different compounds, FT-IR is employed. Figure 2 illustrates the FT-IR spectra of FA (A), P123 (B), and FA-P123 (C) conjugate. By inspecting FA spectrum Fig. 2(A), a strong peak is observed at 1697 cm^−1^; assigned to the –C = O group stretching and a small band at 1607 cm^−1^ resulted from the bending of –NH groups. The IR spectrum of P123 reveals absorbance peaks corresponding to the aliphatic C-H of PEO block stretching and bending vibrations evident at 2887 cm^−1^ and 1460 cm^−1^, respectively. Moreover, the peak at 1112 cm^−1^ is consigned to the stretching vibration of C–O–C of P123. In the P123-FA spectrum, the amide group (-CO–NH-) in the P123-FA structure belonged to the absorption peaks that could be seen between 1,609 and 1,718 cm^−1^ in Fig. 2(C). The FT-IR results permitted the presence of FA in the P123- FA grafting copolymers.

#### ^1^HNMR spectra

**Fig. 3:**
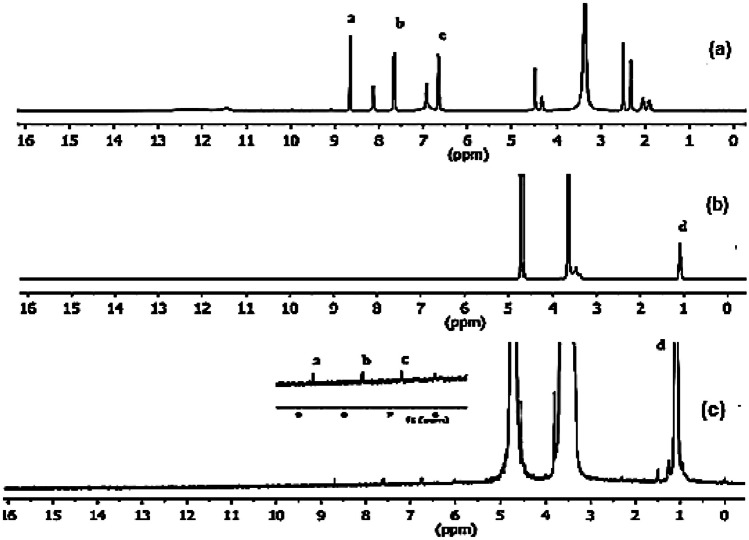
^1^ HNMR spectra of **a **FA, **b **P123 and **c **FA-P123

The ^1^H NMR spectra of P123, FA, and P123-FA conjugate are demonstrated in Fig. 3. The NMR spectrum of P123 reveals a characteristic broad peak at 3.47–3.84 ppm ascribed to –CH_2_CHO segments and –CH_2_CH_2_O–block of PPO and PEO, respectively, and a resonance peak at 1.1 ppm that are distinctive for –CH_3_ groups of PPO. [32]. For the FA spectrum, distinctive resonance peaks at 1.93 ppm, 2.07 ppm, 4.51 ppm, and 8.67 ppm for glutamic acid's (b-CH2), (c-CH2), methylene proton and pteridine proton, respectively were clarified [33]. Considering the P123-FA spectrum, the unique signals from both FA and P123 were illustrated although the FA signals were remarkably weak indicating that it is successfully conjugated with P123 [34].

#### Critical micelle concentration measurement (CMC)

**Fig. 4:**
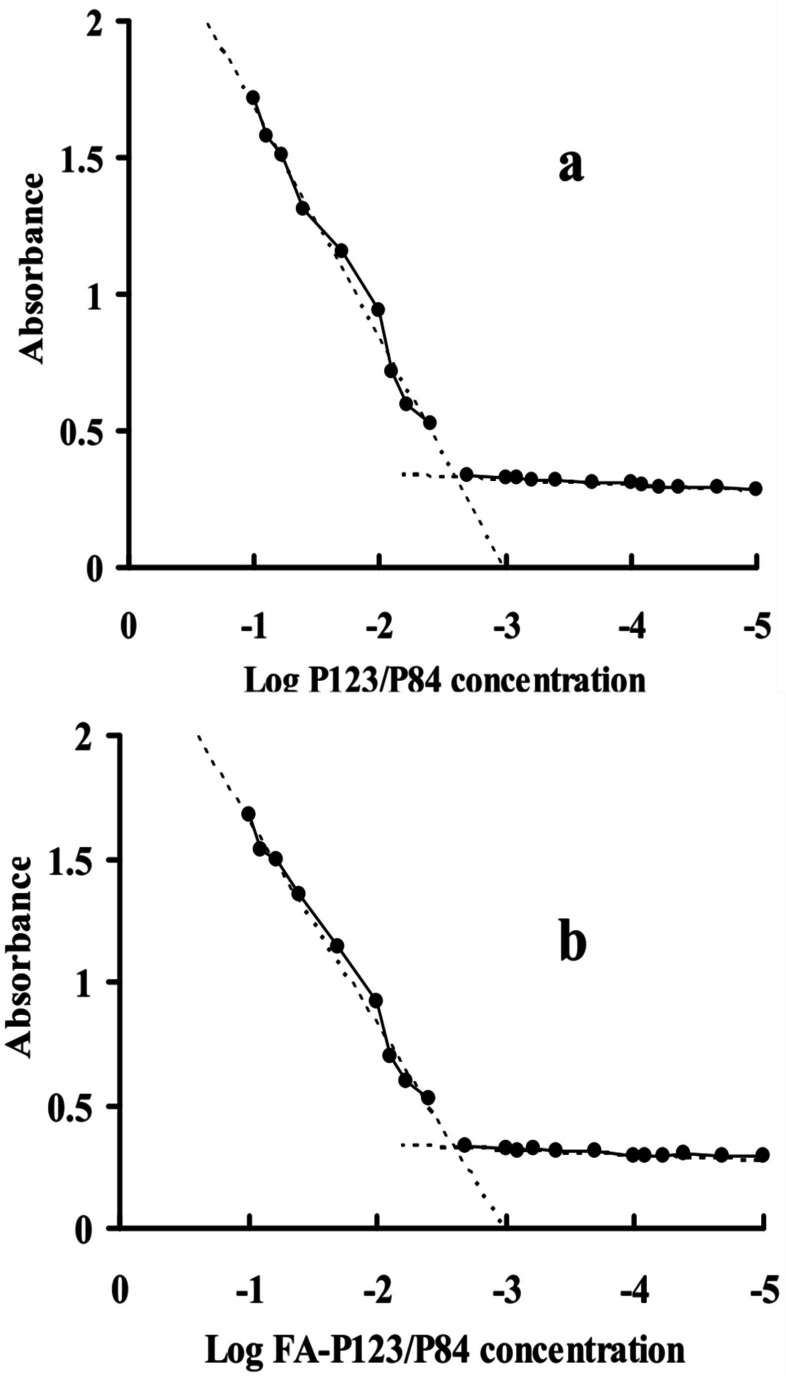
CMC values of P123/P84 (**a**) and FA-P123/P84 (**b**)

Critical micelle concentration (CMC) is an indicator of the stability of drug-loaded polymeric micelles during the in vitro and in vivo study. The stability is an imperative study and depends on the CMC value [35]. In this study, the formation of P123-P84 and FA-P123/P84 mixed polymeric micelles was evaluated by the iodine which was exploited as a hydrophobic probe. Figure 4. displays the relationship between I_2_ absorption intensity and polymer concentration. Whereas the amphiphilic pluronic molecules adjust themselves at a certain concentration, known as the CMC, and isolate the hydrophobic parts from the aqueous environment. These results in the development of colloidal assemblies known as micelles. As revealed in Fig. 4. The CMC of P123-P84 (Fig. 4a) was determined to be (0.00295%) which was like that of FA-P123/P84 (0.0031%) (Fig. 4b). This result proposed that the high stability of polymeric micelles could preserve their integrity even after being heavily diluted in the body and subsequently suggests a longer tour inside the blood supply related to surfactant micelles in vivo [36].

### Characterization of TMXC-loaded mixed polymeric micelles

#### Entrapment efficiency

The EE % of TMXC-loaded mixed polymeric micelles F1 and F2 were 89.09 ± 4.2 and 87.83 ± 5.1%, respectively as revealed in Table 1. The high % EE could be attributed to the large nonpolar head groups of P123 forming a potent hydrophobic core that successfully encapsulate TMXC [37]. The results revealed that the conjugation of FA with P123 had an insignificant impact on the encapsulation efficiency of micelles.

**Table 1:** Physical properties of TMXC-loaded polymeric micelles

**Formula**	**EE%**	**DL%**	**P.S** **(nm)**	**PDI**	**Z.P** **(mv)**
**F1**	89.09 ± 4.2	23.2 ± 0.42	16.63 ± 0.93	0.262 ± 0.032	-15.90 ± 0.84
**F2**	87.83 ± 5.1	25.6 ± 0.43	35.01 ± 1.2	0.280 ± 0.041	-20.5 ± 0.95

#### Particle size, PDI, and surface charge

The polymeric micelle’s size has a significant impact on drug release, stability, and cellular uptake. The mean particle size of mixed polymeric micelles F1 and F2 were 16.63 ± 0.93 nm and 35.01 ± 1.2 nm, respectively as shown in Table 1. From the results, it was observed that particle size for conjugated polymeric micelle (F2) was remarkably higher than the unconjugated one and this was in agreement with [34] findings. Owing to small size FA-conjugated mixed polymeric micelles might exhibit a higher selective accumulation in the solid tumor relying on the presence of folic acid with active targeting ability for folate receptors and passive targeting with EPR effect. This could return to nanoparticles smaller than 200 nm ability to avoid macrophage phagocytosis; prolongs circulation, increases half-lives, and higher accumulation with the solid tumors via a passive target mechanism relying on the enhanced permeation and retention phenomenon [21].

F1 exhibited a negative surface charge (-15.90 ± 0.84 mV) which correlated to the micelle's terminal block of polyethylene oxide (PEO) located on its surface [38], whereas F2 revealed a higher negative value of surface charge (-20.5 ± 0.95 mV), which might be related to the characteristic functional carboxylic group of FA [34]. The high negative surface charge of micelles allows its escaping from clearance by the reticular endothelial system, which subsequently increases the contact time of blood circulation and so accumulates in cancer cells through the EPR effect.

#### In vitro drug release

**Fig. 5:**
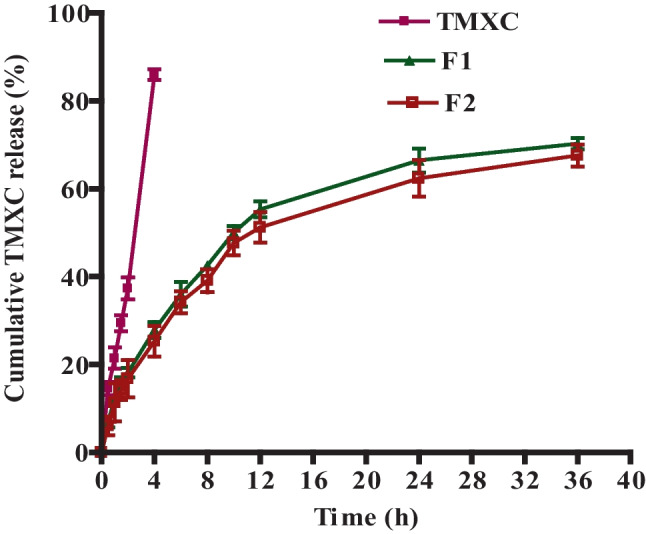
In vitro release profiles of TMXC, F1 (TMXC-loaded P123/P84) and F2 (TMXC-loaded FA-P123/P84) in phosphate buffer saline (pH 7.4) supported with 0.1% polysorbate 80 at 37 °C ± 0.5 °C (n = 3, mean ± SD)

The in vitro drug release of TMXC from the loaded formulae was conducted in phosphate-buffered saline (PBS - pH 7.4), which was supported with 0.1% (w/v) of polysorbate 80 (Fig. 5). TMXC diffused completely through the dialysis membrane in about 4 h. On the other hand, the TMXC release from prepared mixed polymeric micelles F1 and F2 display a biphasic pattern; initial rapid release at the early four hrs. 35% and 30%, respectively followed by sustained release extended for 36 h. The prompt drug release may occur as a result of partial entrapment for loaded TMXC within the external hydrophilic branches of the micelle [39]. However, the delayed sustained release of the drug might come from the expected erosion and erosion of the polymeric micelle that is usually accompanied by a diffusion mechanism that allows for the escape of the drug that is entrapped in the core of the micelle [40]. The diffusion mechanism affords a sustained release of drugs in vivo, enhances their therapeutic efficacy, and diminishes their side effects. Therefore, mixed polymeric micelle carriers have dual effects; maximize TMXC solubility and sustain its release.

Release kinetics models assessment to the drug release data revealed that free TMXC and F1, release obeys Higuchi diffusion based on their regression coefficient values* R*^2^ value of 0.9979. These results may be attributed to the degradation of thin polymeric external branches which allows for drug release in a slow diffusion pattern rather than the erosion pattern [41]. In turn, F2 is best fitted to Korsmeyer-Peppas power with *R*^2^ value of 0.9982. Moreover, the n value obtained was larger than one (1.576) suggesting a super case-II transport, in which several release mechanisms occur, whereas swelling and relaxation are the primary mechanisms that confirm the prolonged drug release and sustain its pharmacological action [42].

#### Stability study

As illustrated in Table 2, TMXC-loaded conjugated micelle (F2) kept its physicochemical properties during storage periods 3 and 6 months at 25 °C without any witnessed changes in the particle size, surface charge, or EE% without any significant changes.

**Table 2:** Effect of storage on physical properties of TMXC-loaded conjugated micelle (F2)

	**Storage periods (months) at 25 ± 2 °C**		
	**0**	**3**	**6**
**EE %**	87.83 ± 5.11	86.50 ± 5.50	85.78 ± 5.45
**P.S (nm)**	35.01 ± 1.20	36.23 ± 1.50	34.90 ± 1.1
**Z.P (mV)**	- 20.50 ± 0.90	-20.80 ± 1.10	- 21.10 ± 0.97

#### In vitro cytotoxicity assessment

**Fig. 6:**
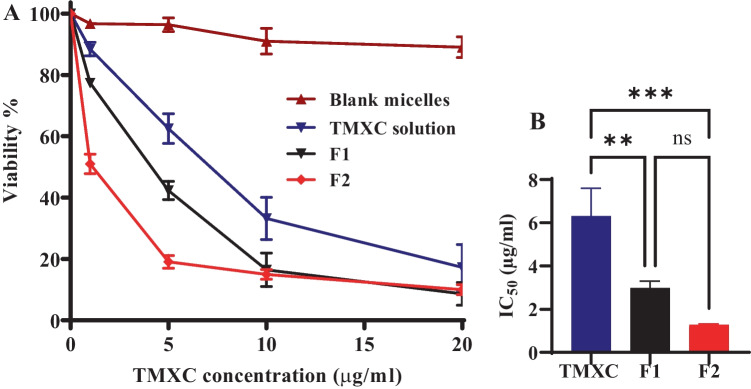
Cell viability assay of blank P123/84 micelle, free TMXC, F1, and F2 (**A**) and IC_50_ values of free TMXC, F1, and F2 (**B**) against MCF-7 cell line. The degree of significance for the given comparison is represented by the number of symbols, **p < 0.01, ***p < 0.001, and ns = not significant

The human breast cancer cell line (MCF-7) was selected for assessment of in vitro antitumor activity of TMXC, mixed polymeric micelles F1 and F2 via MTT assay. The additive value of the mixed polymeric micelles and FA conjugation with TMXC were measured by the determination of the IC_50_ value; which represents the drug concentration required to prevent 50% of the growth of incubated cells within a predetermined time interval [43]. Figure 6. demonstrates the cell viability findings achieved using MCF-7 cells after being exposed for 72 h. The blank polymeric micelles did not display any substantial cytotoxicity against MCF-7 cells, whereas the IC_50_ of free TMXC (6.30 µg/ml) was significantly decreased to 2.98 µg/ml and 1.28 µg/ml for F1 and F2 micelles, respectively. However, the difference between IC_50_ values of F1 and F2 was not statistically significant (Fig. 6B). This could be attributed to the higher solubility of TMXC which was stabilized in the deep core of the micelle resulted in more elevated targeting to tumor cells through the cellular leak of the controlled released drug release [21]. Additionally, the elevated cytotoxicity of conjugated micellefs could be attributed to FA located on the surface of the tested cancer cell line [44]. This finding prompts the interaction between the folate receptors on the MCF-7 cells and folate located on the micelle surface via a folate receptor-mediated mechanism resulting in successful uptake by MCF-7 cells producing higher cytotoxicity [45]. Therefore, more drug was incorporated into the tumor cells to accomplish a better anticancer effect.

#### In vitro cellular uptake study

**Fig. 7:**
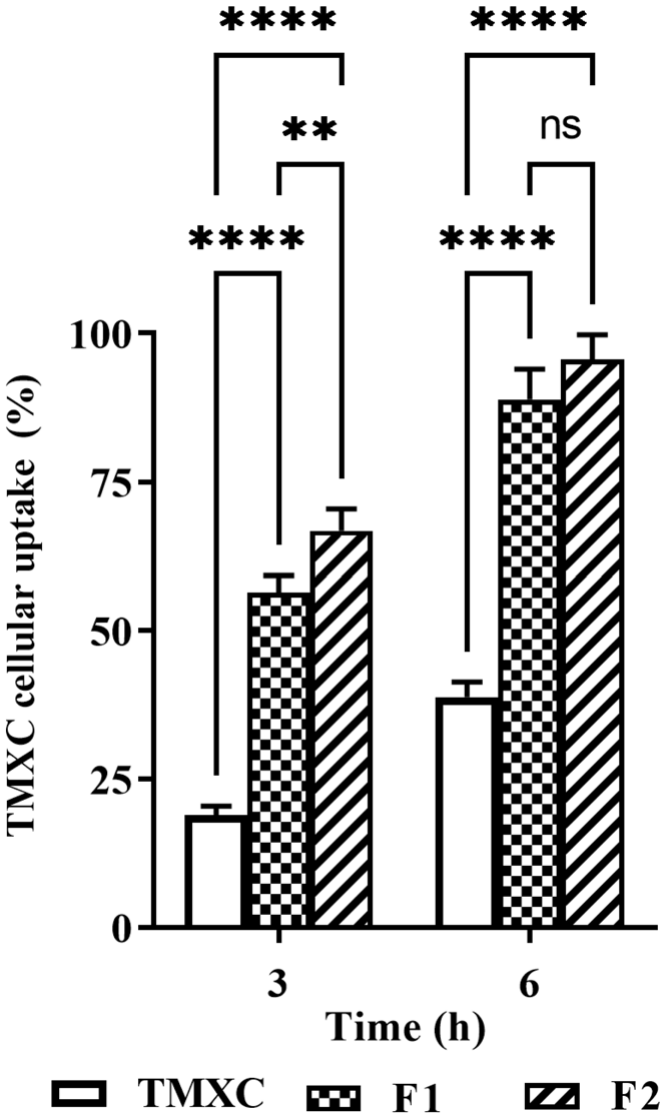
In vitro cellular uptake behavior for free TMXC, F1, and F2 with MCF-7 tumor cells. The degree of significance for the given comparison is denoted by the number of symbols, **p < 0.01, ****p < 0.0001, and ns = not significant

This study was conducted to evaluate the influence of the conjugated FA-P123/84 micelles (F2) on the cellular uptake of TMXC compared to unconjugated micelles (F1) and free TMXC (Fig. 7). The results revealed that, after 3 and 6 h, both F1 and F2 exhibited 2.28 and 2.48-fold higher values of TMXC cellular uptake % compared to free TMXC, respectively. Statistical analysis indicated that, after 3 h, F2 exhibited a significantly higher % of cellular uptake compared to free TMXC and F1. Also, a statistically significant difference between F1 and F2 was observed. While after 6 h, F2 achieved a significantly (p < 0.0001) higher cellular uptake % over free TMXC and a slightly higher cellular uptake than F1, but the difference between F1 and F2 was not statistically significant. The higher cellular uptake of conjugated micelles could be attributed to the interaction between the folic acid located on the micelle surface and folate receptors on the MCF-7 cells via a folate receptor-mediated mechanism. These findings are consistent with those of the in vitro anticancer activity assay and indicated that polymeric nano micelles based on FA as a targeted ligand are assumed to be a promising delivery system for anticancer drugs [33].

#### In vivo antitumor effect

**Fig. 8:**
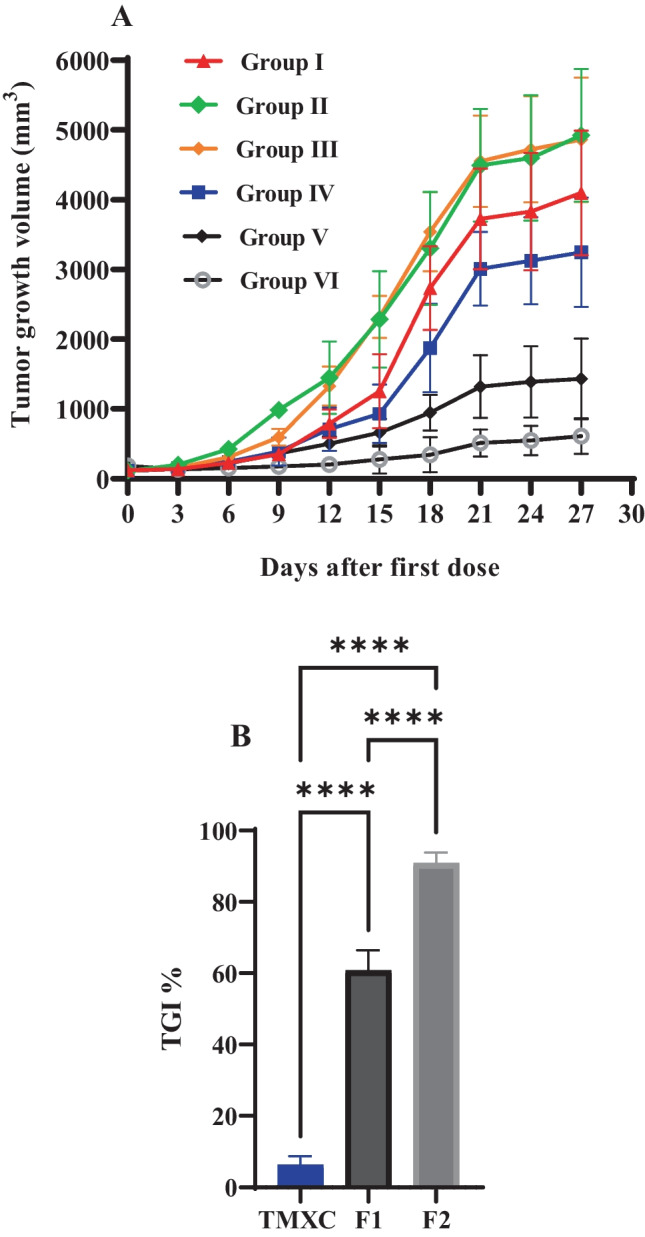
Tumor growth volume of mice groups (**A**); group I (control), Group II (Plain P23/P84), group III (Plain FA-P123/P84), group IV (TMXC), group V (TMXC-loaded P123/P84) and group VI (TMXC-loaded FA P123/P84) (n = 12) and TGI % in groups receiving treatments containing TMXC (**B**). ****p < 0.0001

Tumor-bearing Swiss albino mice were used as an established model for the assessment of the antitumor activity of TMXC-loaded polymeric micelles versus breast cancer growth [46]. The tumor volume and the percentage of tumor growth inhibition (TGI %) were the indicators for the relative anticancer effectiveness. The results revealed the quick tumor growth in control group which received an isotonic saline (Fig. 8). Meanwhile, TGI% in groups treated with blank micelles, group II and group III, were -31.26% and - 6.20% respectively at the end of the experiment which indicates the absence of cytotoxicity of polymeric micelle and folic acid as a targeting linker and negligible antitumor activity of plain formulae [34]. On the other hand, the calculated TGI % in groups treated with free TMXC, F1 and F2 was increased to 10.12, 60.77, and 90.86% respectively. Additionally, TMXC-loaded FA P123/P84 micelles (F2) showed significantly (p < 0.0001) higher TGI % compared to either free TMXC or TMXC-loaded P123/P84 (F1) micelles (Fig. 8B). These results proved that the antitumor activity of TMXC-loaded FA-P123/P84 micelles is probably due to the combination of two potential mechanisms: (a) Enhanced Permeability and Retention (EPR) effect; (b) selective uptake by cancer cells through receptor-mediated endocytosis. The EPR effect was interceded by the small size of polymeric micelles encouraging the passive permeability at the tumor site. The receptor-mediated endocytosis was predominantly due to the existence of folate moiety on FA-P123/P84 micelles. Also, interstitial accumulation in solid tumors augments their anticancer effects through localization and declines their systemic side effects [47].

##### Effects on body weight

**Fig. 9:**
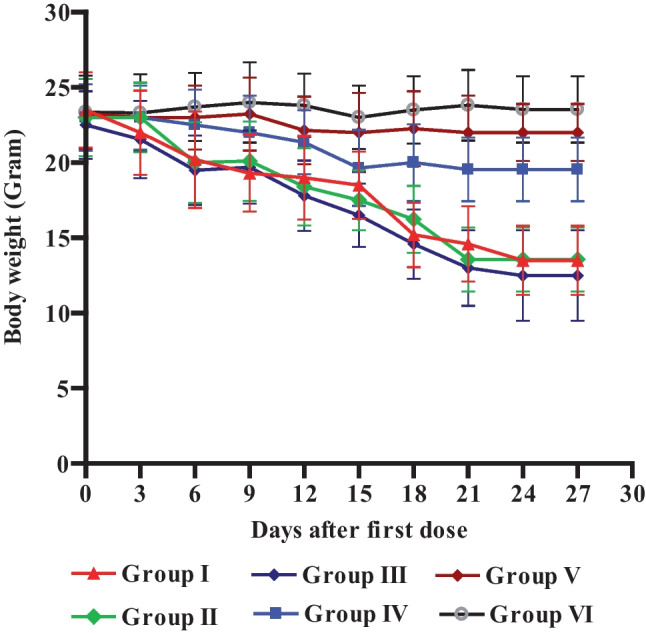
Body weight change in mice groups; group I (control), group II (Plain P123/P84), group III (Plain FA-P123/P84), group IV (free TMXC), group V (TMXC-loaded P123/P84) and group VI (TMXC-loaded FA-P123/P84) (the results represent the mean ± SD, n = 12)

Mice received plain micelles (groups II and III) presented a significant loss in their body weights compared to groups IV, V, and VI (Fig. 9). Simply, the mice’s weight dropped from 23 to 13.56 g and from 22.5 to 13 g in groups II and III, respectively over the period of treatment. However, in group IV treated with pure TMXC; the weight decreased from 24 to 19.55, while declined from 24.2 to 21.2 g and from 24.35 g to 23.82 g with mice treated with both groups V and VI respectively. These results intend that TMXC and its formulated micelles did not alter energy intake and might increase energy spending instead [48].

##### TMXC biodistribution in tumor tissues

**Fig. 10:**
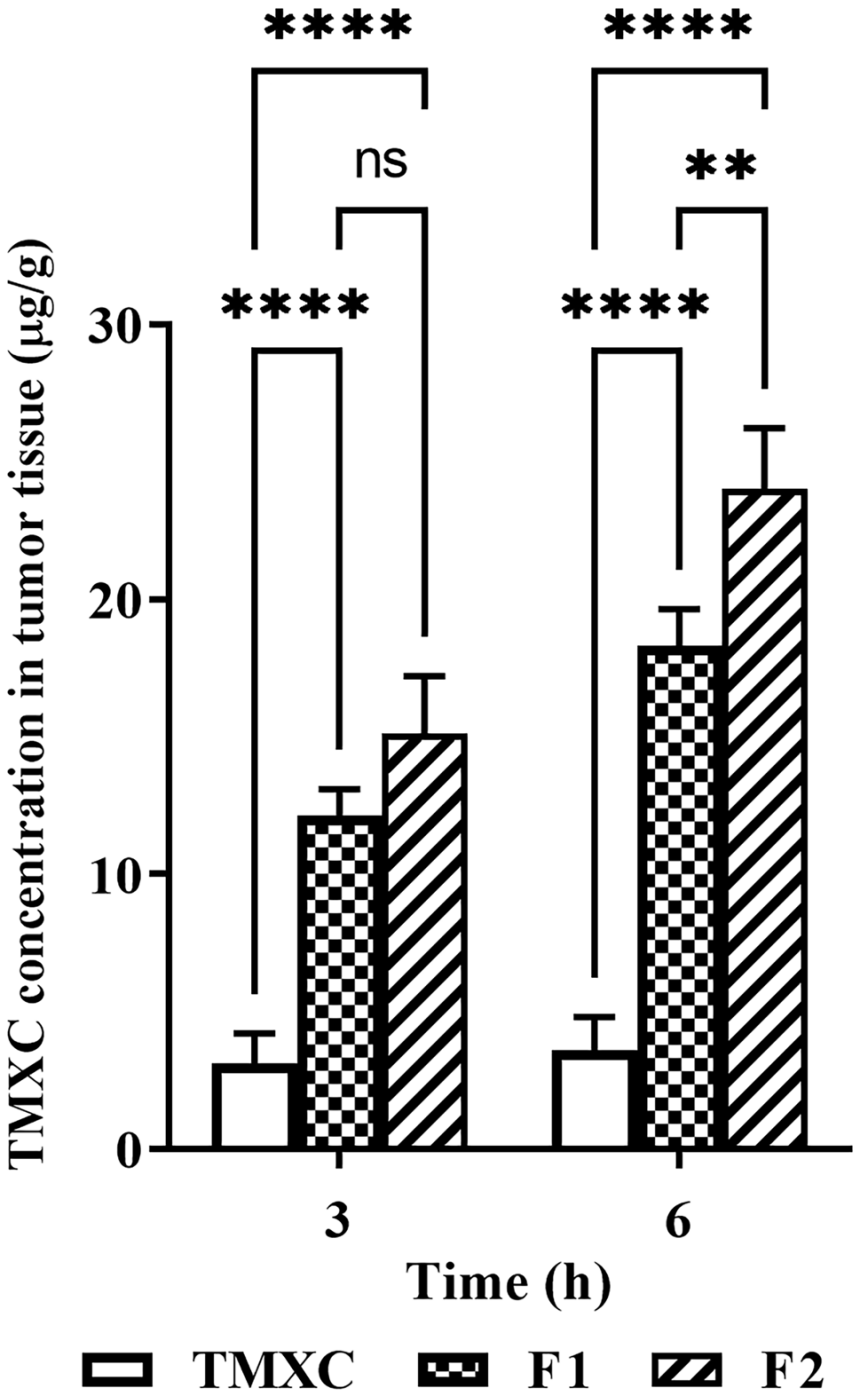
In vivo tumor tissue distribution of the TMXC in Swiss Albino mice after tail vein injection of free TMXC, F1, and F2 (n = 6). **p < 0.01, ****p < 0.0001, and ns = not significant

At the end of the previous experiment, TMXC level in mice’s solid tumor samples was determined after tail vein injection of F1, F2, and free TMXC suspension to endorse the cancer localization. Results exhibited the ability of F2 as a promising carrier for targeting solid tumors which increases the retention of TMXC within the tumor tissues Fig. 10. The obtained results indicated a significantly higher TMXC concentration within the tumor tissue for F2 over the free TMXC after either 3 or 6 h. Also, our findings revealed a significant accumulation in the case of F2 over F1 after 6 h, but after 3 h, the difference between F1 and F2 was statistically insignificant. The obtained results were convenient with our previous hypothesis which suggested that the conjugation of our previously prepared polymeric micelles [9] with folic acid as a good linker for folate receptors might be an effective way for drug targeting and tumor localization. The drug accumulation within the tumor tissues is a result of the interaction between two potential mechanisms: passive targeting through the EPR phenomenon and active targeting through receptor-mediated endocytosis [49]. The tiny size of polymeric nanomicelles promoted passive permeability and tumor localization, which in turn facilitated the EPR effect. Instead of passive diffusion through the cell membrane, F2 may be absorbed into the intended cell after attaching to the cell surface via a folate receptor-mediated endocytosis. Higher TMXC concentration would therefore be obtained inside the cell, better inhibiting tumor development [47].

### Histopathological assay

**Fig. 11:**
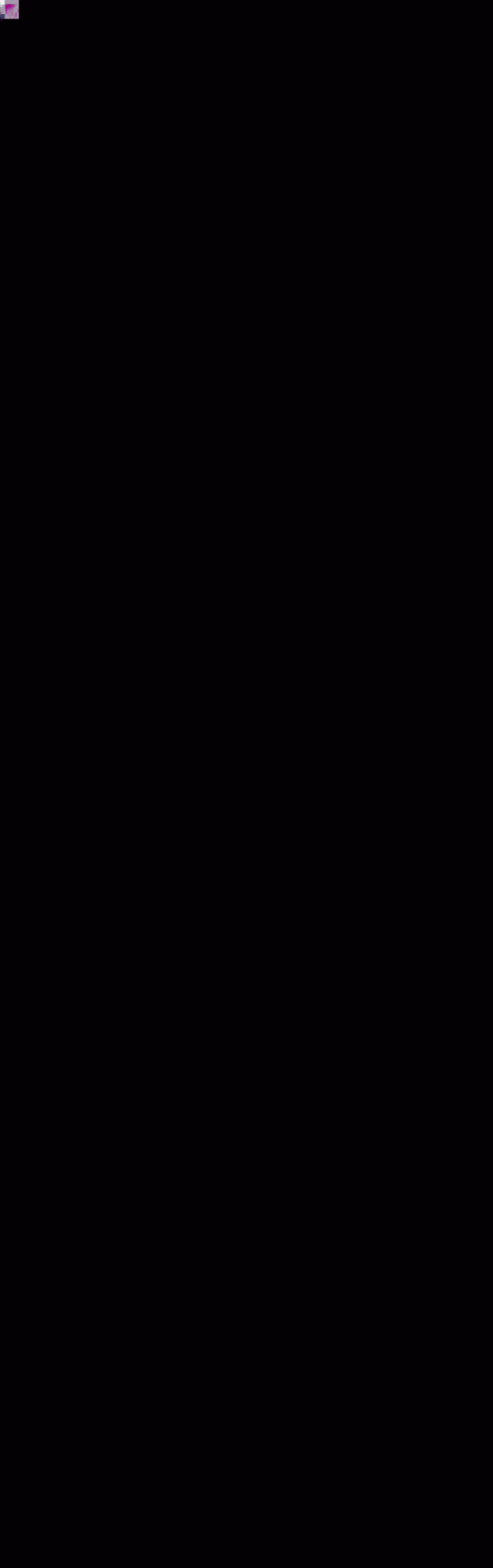
Histopathological sections in Ehrlich solid tumor for control group I: presented numerous intact anaplastic cells in most areas of the muscle (M); group IV: few necrosis and apoptosis in few cells with intact muscle (M, N); group V: moderate necrosis and apoptosis cells (N); group VI: Remarkable increase in necrosis and apoptosis cells (A, N)

Histopathological examination of mice Ehrlich solid tumor differentiates the quantity of necrosis and apoptosis cells presented in treated and untreated groups Fig. 11. For the control group, numerous intact anaplastic cells (C) (free from necrosis and apoptosis) in most areas of the muscle (Ms) with criteria of anaplasia and hyperchromachia (H) as polarity, pleomorphic and mitotic activity were clarified. However, by observing mice treated with free TMXC (group IV) tumor cells as well as polymeric micelles group V and VI; necrosis and apoptosis in a few cells with intact muscle were gradually varied. In group IV, few necrosis and apoptosis in a few cells with intact muscle were detected. While for mice treated with unconjugated polymeric micelle group V; necrosis and apoptosis increased in some cells gradually. Moreover, a remarkable increase in the degree of necrosis and apoptosis was noticed in most tumor cells for mice treated with conjugated micelles group VI. These investigations confirmed the polymeric micelles and FA attribution to the defective apoptosis enhancement [50].

## Conclusions

Our findings explored the perspective of TMCX-loaded FA mixed polymeric micelles (F2) as an effective delivery system for breast cancer therapy. TMXC-loaded FA-P123/P84 micelles were successfully prepared using carbonyl diimidazole cross-linker chemistry. F2 exhibited small size and maximum encapsulation efficiency displaying biphasic drug release modality, with initial burst release followed by prolonged effect over 36 h. Moreover, owing to the targeting properties of FA, the polymeric micellar system could amplify cellular uptake inside the tumor tissue with elevated cytotoxicity compared to pure TMXC. Besides, a high value of TGI % obtained with conjugated micelles proved the improved therapeutic impact and anticancer performance of TMCX encapsulated into polymeric micelles core. Additionally, the in vivo tumor localization obtained through (passive and active targeting) as well as the remarkable increases in the degree of necrosis and apoptosis of tumor cells adapted them to be as smart nano-size targeted candidates for high-dose delivery of TMCX into cancer cells in talented and safe pathway.

## Data Availability

The datasets generated during and/or analyzed during the current study are available from the corresponding author upon reasonable request.
